# Identification of Novel Genomic Aberrations in AML-M5 in a Level of Array CGH

**DOI:** 10.1371/journal.pone.0087637

**Published:** 2014-04-11

**Authors:** Rui Zhang, Ji-Yun Lee, Xianfu Wang, Weihong Xu, Xiaoxia Hu, Xianglan Lu, Yimeng Niu, Rurong Tang, Shibo Li, Yan Li

**Affiliations:** 1 Department of Hematology, The First Affiliated Hospital of China Medical University, Shenyang, Liaoning, P.R. China; 2 Department of Pediatrics, University of Oklahoma Health Sciences Center, Oklahoma City, Oklahoma, United States of America; 3 Department of Pathology, College of Medicine, Korea University, Seoul, South Korea; University of Navarra, Spain

## Abstract

To assess the possible existence of unbalanced chromosomal abnormalities and delineate the characterization of copy number alterations (CNAs) of acute myeloid leukemia-M5 (AML-M5), R-banding karyotype, oligonucelotide array CGH and FISH were performed in 24 patients with AML-M5. A total of 117 CNAs with size ranging from 0.004 to 146.263 Mb was recognized in 12 of 24 cases, involving all chromosomes other than chromosome 1, 4, X and Y. Cryptic CNAs with size less than 5 Mb accounted for 59.8% of all the CNAs. 12 recurrent chromosomal alterations were mapped. Seven out of them were described in the previous AML studies and five were new candidate AML-M5 associated CNAs, including gains of 3q26.2-qter and 13q31.3 as well as losses of 2q24.2, 8p12 and 14q32. Amplication of 3q26.2-qter was the sole large recurrent chromosomal anomaly and the pathogenic mechanism in AML-M5 was possibly different from the classical recurrent 3q21q26 abnormality in AML. As a tumor suppressor gene, *FOXN3*, was singled out from the small recurrent CNA of 14q32, however, it is proved that deletion of *FOXN3* is a common marker of myeloid leukemia rather than a specific marker for AML-M5 subtype. Moreover, the concurrent amplication of *MLL* and deletion of *CDKN2A* were noted and it might be associated with AML-M5. The number of CNA did not show a significant association with clinico-biological parameters and CR number of the 22 patients received chemotherapy. This study provided the evidence that array CGH served as a complementary platform for routine cytogenetic analysis to identify those cryptic alterations in the patients with AML-M5. As a subtype of AML, AML-M5 carries both common recurrent CNAs and unique CNAs, which may harbor novel oncogenes or tumor suppressor genes. Clarifying the role of these genes will contribute to the understanding of leukemogenic network of AML-M5.

## Introduction

Acute monocytic leukemia, also named acute myeloid leukemia-M5 (AML-M5), is one of the most common subtypes of AML defined by the French-American-British (FAB), which is comprised by more than 80% of monoblasts (AML-M5a) or 30–80% monoblasts with (pro)monocytic differentiation (AML-M5b). Responses to chemotherapy and prognosis in AML-M5 patients are varied. The recently proposed World Health Organization (WHO) classification attempts to associate the prognostic variance with cytogenetic abnormalities [1]–[2]. However, rather than t(8;21) in AML-M2, t(15;17) in AML-M3 and inv(16)/t(16;16) in AML-M4eo, specific subtype-associated translocation is lacking in AML-M5. It has been described that a part of AML-M5 patients carry 11q23 (MLL gene) rearrangement with a wide spectrum of recurrent translocation partner chromosomes [3]–[4]. In this sense, it is reasonable to assume that unbalanced chromosomal abnormalities might play a major role in the leukemogenesis of AML-M5. Especially those cryptic alterations, which are invisible for traditional banding analysis, are not rare in AML with normal karyotype [5]–[6].

It is necessary to evaluate the chromosomal changes in a whole genomic level. However, banding analysis, currently as the first choice to evaluate the genomic abnormalities of AML, is frequently to get inconclusive results due to chromosome condensation, imperfect banding, and the presence of few metaphases. FISH is a more sensitive technique, but is limited by the genomic coverage properties. Array CGH provides some distinct advantages including the potential for comprehensive genomic profiling and the ability to delimit the boundaries of specific genomic aberrations. Recently, it is considered as a powerful method to analyze genomic imbalances in hematopoietic malignancies, especially to determine cryptic and recurrent anomalies not observed by routine techniques [7]–[11].

The primary aim of present study is to assess, by means of array CGH assay, the possible existence of unbalanced chromosomal abnormalities, and delineate the copy number alterations (CNAs) in 24 cases of adult AML-M5 patients. Besides this, recurrent CNAs harboring cancer genes are also analyzed. The novel candidate oncogenes and tumor suppressor genes aberrations which might be implicated in the leukemogenesis of AML-M5 were revealed in this study.

## Results

### Normal karyotype was in the majority of AML-M5 cases

Karyotypes were available for 16 AML-M5 cases (Table S1 in File S1). 11 of them (10 newly diagnosed cases and 1 relapsed case) had normal karyotypes and 5 cases carried 2 or 3 chromosomal abnormalities. t(9;11)(p22;q23) as the recurrent chromosomal change was presented in 3 cases.

### Array CGH delineated the genomic imbalances of AML-M5

**Figure 1:**
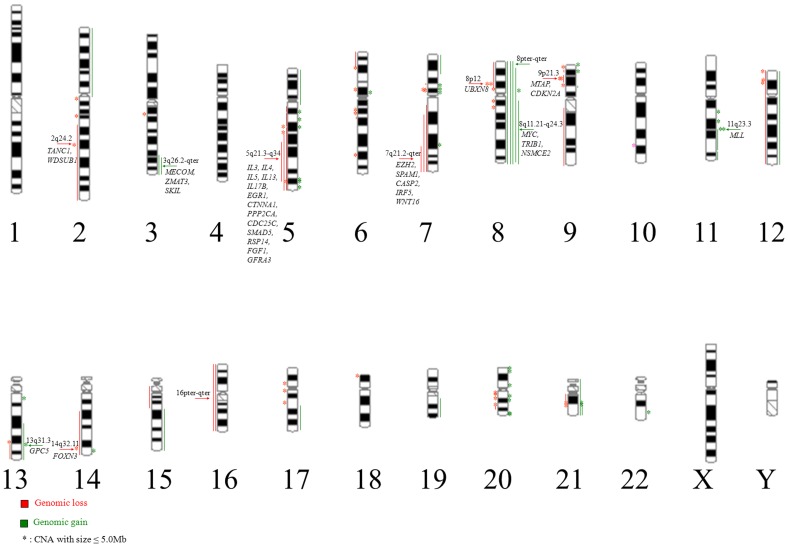
Distribution of CNAs recognized by array CGH in 24 patients with AML-M5. A total of 117 CNAs with 51 genomic gains (43.1%) and 66 genomic losses (56.9%) was observed. 70 CNAs (59.8%) were cryptic with size less than 5 Mb and invisible to karyotype. The arrows indicated recurrent CNAs and harbored interesting genes.

A total of 117 CNAs with size ranging from 0.004 to 146.263 Mb were recognized in 12 of 24 cases, involving all chromosomes other than chromosome 1, 4, X and Y (Figure 1 and Table S1 in File S1). The cryptic chromosomal aberrations less than 5 Mb took up 59.8% of all CNAs (Figure 1). It was worth noting that in the 10 newly diagnosed cases with normal karyotype, microduplications spanning 11q23.3 carrying *MLL* and 5q35.3 carrying *AGXT2L2* and *COL23A1* were detected in case #8 and case #16, respectively. 12 recurrent CNAs were mapped in Figure 1 and listed in Table 1. Notably, in contrast to the most common recurrent CNAs reported by 8 representative genomic studies of MDS and AML using oligonucleotide array CGH or SNP array [5], [12]–[18] (Table 2), novel CNAs including gains of 3q26.2-qter and 13q31.3 as well as losses of 2q24.2, 8p12 and 14q32 were proposed in the present study. Except that gain of 3q26.2-qter is a large chromosomal abnormality, all the other recurrent CNAs were cryptic and harbored interesting genes.

**Table 1:** The recurrent chromosomal abnormalities of 24 cases of AML-M5.

Chromosome Region	Genomic coordinates (NCBI Build 36.3)	Size(Mb)		Number of cases (case #)	Interesting genes
		Gain	Loss		
2q24.2*	159,772,558→159,847,666		0.075	2 (#11, #13)	*TANC1, WDSUB1*
3q26.2-qter*	170,262,958→199,381,714	29.119		2 (#12, #18)	*MECOM, ZMAT3, SKIL, SOX2, EVI1*
5q21.3-q34	104,803,921→163,613,351		58.809	3 (#7, #11, #19)	*IL3, IL4, IL5, IL13, IL17B, EGR1,CTNNA1, PPP2CA, CDC25C, SMAD5, RSP14, FGF1, GFRA3*
7q21.2-qter	92,258,287→158,816,03		66.558	3 (#7, #11, #12)	*EZH2, SPAM1, CASP2, IRF5, WNT16*
8p12*	30,721,389→30,765,786		0.044	3 (#11, #13, #18)	*UBXN8*
8q11.21-q24.3	50,233,257→142,198,258	91.965		2 (#11and #24)	*MYC,TRIB1, NSMCE2*
8pter-qter	71,702→146,264,232	146.257		3 (#14, #17, #19)	*N/A*
9p21.3	21,819,027→21,986,390		0.167	3 (#7, #13, #18)	*MTAP, CDKN2A*
11q23.3	117,844,729→117,849,872	0.004		3 (#7, #8, #18)	*MLL*
13q31.3*	90,024,777→91,278,708	1.254		2 (#11, #17)	*GPC5*
14q32.11*	89,027,534→89,236,808		0.209	2 (#13 and #24)	*FOXN3*
16pter-qter	18,227→88,715,037		88.802	2 (#7 and #19).	*N/A*

N/A: not applicable;

*recurrent CNA first rep°rted by this study.

**Table 2:** Recurrent CNAs reported by previous studies of MDS/AML and this study.

Genomic aberrations	Common recurrent CNAs of MDS/AML*	Recurrent CNAs of AML-M5 in this study
Gain	8q24.13-q24.21(*MYC*), 11q23.3(*MLL*), 12p13.32(*CCND2*), 21q22.2(*ERG, TMPRSS2*)	3q26.2-qter, 8q11.21-q24.3, 8pter-qter, 11q23.3, 13q31.3
Loss	5q, 7q, 9p21.3(*CDKN2A*), 12p13.31-p13.2(*ETV6*), 16q22.1(*CBFB*), 17p13(*TP53*), 17q11.2(*NF1*), 18p11.2(*PTPN2*), 21q22.12(*RUNX1*)	2q24.2, 5q21.3-q34, 7q21.2-qter, 8p12, 9p21.3, 14q32.11, 16pter-qter

*: data based °n eight representative genomic studies of MDS and AML using oligonucleotide array CGH or SNP array.

### Translocation and inversion rather than partial duplication of 3q26.2 were identified by FISH

By array CGH, 3q26.2-qter was identified as a recurrent chromosomal anomaly. To investigate whether the gain of 3q26.2-qter, was closely associated with AML-M5 or other subtypes of AML, FISH with EVI (3q26.2) break-apart probe was hybridized in bone marrow cells of 69 cases of de novo AMLs (Table S2 in File S1). Normal two fusion signals were observed in 67 cases and abnormal FISH signal pattern with two fusion signals plus an extra red signal (TEL'EVI1) was shown in another two cases. One of the cases (ID: 37) was AML-M5, containing 89% of abnormal cells in the interphase. t(3;21) in the metaphase were proven by subsequent FISH with ETV6/AML1 probe (Figure S1A–F in File S1). The other one (ID: 65) was AML-M6, which carried 93% of abnormal cells produced by inv(3)(p25;q26) (Figure S1G–I in File S1). No partial duplication of 3q involving EVI gene was observed.

### Candidate oncogenes and tumor suppressor genes were inferred by array CGH

**Figure 2:**
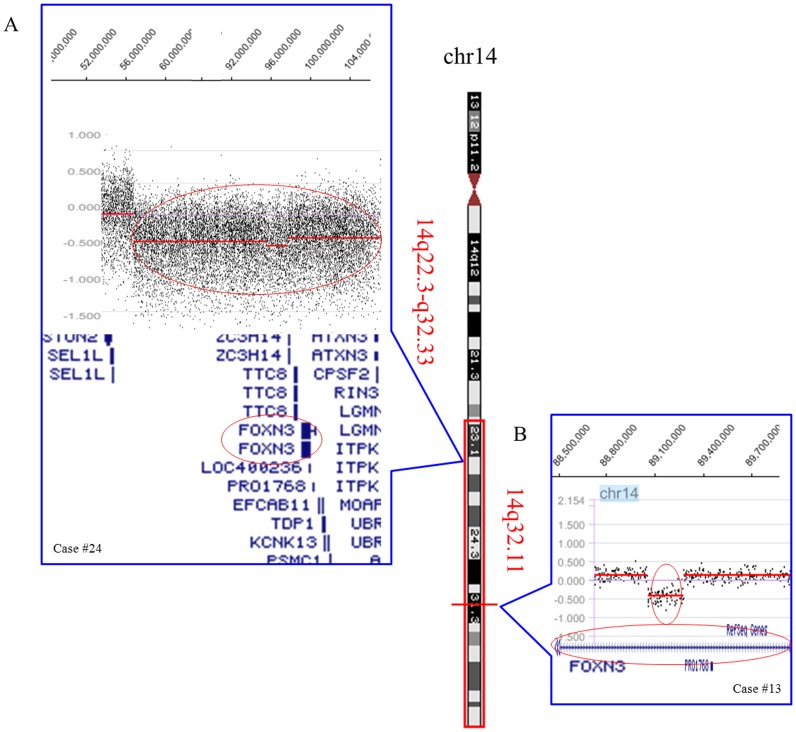
*FOXN3* gene (14q31.3-q32.11) deletions in two patients with AML-M5. (A) Array CGH showed a lower signal ratio spanning 14q22.3-q32.33 (55,109,046–106,342,076 bp). *FOXN3* gene was one of the genes located at the deleted region. (B) The microdeletion of 14q32.11 (89,027,534–89,236,808 bp) involving *FOXN3* gene was inferred by array CGH.

**Figure 3:**
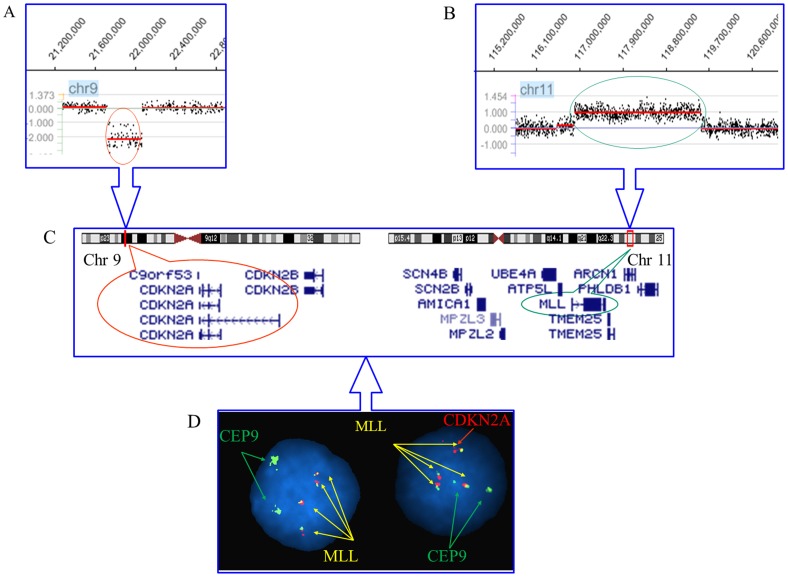
Gain of 11q23.3 and loss of 9p21.3 coexisting in case #18. (A) Lower signal of 21,755,226–22,097,452 on chromosome 9 was recognized by array CGH analysis. (B) A gain in the region of 116,893,506–119,524,806 was observed on chromosome 11 by array CGH. (C) UCSC genome browser (hg18) indicated the RefSeq genes involved *CDKN2A/B* genes and *MLL* gene. (D) FISH analysis showed the concurrent *CDKN2A* gene deletion and *MLL* gene amplication in interphase nuclei. The left nucleus showed the homogenous deletion of *CDKN2A* and the right nucleus demonstrated the heterogenous deletion of *CDKN2A*.

Oncogenes and tumor suppressor genes that have been reported to be implicated in carcinogenesis or leukemogenesis were further singled out from the cryptic recurrent CNAs (Table 3). Microdeletion of 0.209 Mb and large terminal deletion of 51.233 Mb encompassing *FOXN3* gene at 14q were observed in two cases (case #13 and #24) (Figure 2). We subsequently investigated the expression levels of the *FOXN3* in a cohort of acute leukemia samples including 78 AMLs and 19 ALLs (Table S3 in File S1). In comparison with healthy controls (HCs) (1.80±0.27), significantly lower expression of *FOXN3* was found in the AML group (0. 90±0.12) (*p*<0.05) (Figure S2 in File S1). No significant difference was observed between AML-M5 and non-AML-M5 (Figure S3 in File S1). A low tendency toward expression of *FOXN3* was detected in the ALL group (1.16±0.65), but the expression was not significantly different from the controls nor the AML groups (*p*>0.05) (Figure S2 in File S1). Gain of 11q23.3 containing *MLL* gene was observed in 3 cases with size ranging from 0.004 Mb to 23.133 Mb. Cryptic chromosomal losses of 9p21.3 or a large deletion of 9p involving *CDKN2A* and *MTAP* were observed in 3 cases. Strikingly, gains of 11q23.3 and losses of 9p21.3 coexisted in two cases. To confirm the concurrent deletion of 9p21.3 and amplication of 11q23.3 inferred by array CGH, FISH analysis using *MLL* and *CDKN2A* probes was performed on case #18 (Figure 3). Consistent with array CGH, amplication of *MLL* with three or four fusion signals was shown in 69.5% of the 200 nuclei. Homogenous and heterogenous deletion of *CDKN2A*, indicated by loss of double or single red signal, was observed in 71.5% and 19% of 200 examined nuclei respectively. Totally, *MLL* amplication and *CDKN2A* deletion concurred in 68.5% of 200 nuclei.

**Table 3:** Candidate AML-M5 associated genes inferred by array CGH.

Genomic aberrations	Gene symbol	Gene name	Chromosome location	Function
Gain	*MLL*	mixed-lineage leukemia	11q23.3	Regulation of hematopoiesis
Loss	*CDKN2A*	Cyclin-dependent kinase inhibitor 2A	9p21.3	Cell cycle regulation, tumor suppressor
	*MTAP*	Methylthioadenosine phosphorylase	9p21.3	Deficient in many cancers because this gene and the tumor suppressor *CDKN2A* gene are co-deleted.
	*FOXN3*	Forkhead box N3	14q31.3-q32.11	Cell cycle regulation

### Correlation of CNAs number and clinico-biological parameters

To investigate the prognostic value of the number of array CGH aberrations, 22 cases of newly diagnosed AML-M5 were grouped based on the numbers of CNA. As the median of CNAs per case was four, cases with four or less CNAs were grouped apart from cases with five or more. The numbers of CNA in the two groups were correlated with clinico-biological parameters which suggest a poor prognosis. These parameters included age>60 years old, white blood cell (WBC) count >10×10^9^/L, hemoglobin concentration <80 g/L, platelet count <50×10^9^/L, higher percent of blast cells in bone marrow (comparing to the average blast ratio in bone marrow) and responses to treatment, such as number of CR. By statistic analysis, no significant difference was observed between the two groups (table 4).

**Table 4:** Comparative clinico-biological parameters and number of CR by number of CNAs.

Number of CNA	Total number	Age>60(ys)	WBC>10×10^9^/L	Hb<80 g/L	PLT<50×10^9^/L	Blast%>0.72 in bone marrow	Number of CR
≤4 CNAs	17	2	12	6	9	11	8
>5 CNAs	5	2	5	2	2	3	1
p value	–	0.2089	0.2899	1.0000	1.0000	1.0000	0.3602

## Discussion

In the last few years, the classification of AML has developed from a single morphological level to a MICM basis. As one of the diagnostic methods, genetic abnormality examination plays an important role in further sub-classification of AML and gives more clues to therapy selection and prognosis [1]–[2]. The cohort of patients of our study is in a special subgroup of AML, AML-M5, which is characterized by variable therapy responses and low incidence of recurrent chromosomal abnormalities at the level of conventional cytogenetic method. The present study refined the genomic aberrations of AML-M5 by an integration of chromosome, FISH and array CGH procedure. The majority of the genomic alterations are cryptic and invisible to G-banding analysis, indicating that high-resolution array CGH approach is necessary to provide more precise description of chromosomal aberrations in AML-M5. Particularly, in the patients with normal karyotype, array CGH has been able to unveil the small CNAs. However, similar with the previous studies, the recurrence of the small CNAs is very low [19]–[20]. Normal genomes are observed in nearly half of the AML-M5s, inferring that additional studies, such as gene sequencing or epigenetic analysis, might be necessary to investigate the underlying mechanism of AML-M5.

Array CGH improves the detectable rate of chromosomal abnormalities, which make it easier to map the recurrent chromosomal aberrations of AML-M5. As a subtype of AML, it seems that AML-M5 carries both the common recurrent CNAs which have been reported in the previous AML studies and novel recurrent CNAs which are noted as candidate AML-M5 associated CNAs. However, whether these novel recurrent CNAs are specially associated with AML-M5 are needed to be clarified. Gain of 3q26.2-qter is the sole large recurrent chromosomal change in the present study. There is an overlap between 3q26.2-qter in case 12 and 3q21.1-qter in case 18. It is known that 3q21q26 anomaly is the most common karyotype in acute myeloid patients with 3q abnormalities [21]–[22]. *RPN1* gene at the breakpoint of 3q21.3 juxtaposing to *EVI1* gene at the breakpoint of 3q26.2 is the key mechanism of this type of AML. However, the significance of 3q26.2-qter amplication is not well known in AML although it is considered as a recurrent chromosomal abnormality in several solid tumors [23]–[25]. This study inferred a possible recurrent amplication of 3q26.2-qter, which is associated with *EVI* gene by array CGH. However, neither its recurrence in AML nor its specific association with AML-M5 was confirmed by FISH. There's limitation on the confirmatory FISH analysis since it only focus on *EVI* gene. Recent study by Hussenet and his colleagues reported that *SOX2* is an oncogene activated by recurrent 3q26.3 amplifications in human lung squamous cell carcinomas [26]. Therefore, amplication of 3q26.2-qter might serve as a novel recurrent 3q abnormalities and it possibly has a different leukemogenic mechanism from the classical 3q21q26 anomaly in AML.

By the advantages of array CGH, the candidate genes in the cryptic recurrent CNAs have been singled out. It is the first time that *FOXN3* deletion is observed in leukemia. The *FOXN3* acts as a transcriptional repressor and has been implicated in DNA damage-inducible cell cycle arrests in saccharomyces cerevisiae [27]. However, the physiological functions of *FOXN3* in mamals are not known. Reduced expression of *FOXN3* has been reported in several cancers, such as renal cell carcinoma and oral squamous cell carcinoma [28]–[29]. Scott *et al*. found that *FOXN3* recruited SKIP, which played a role in cell growth and differentiation through the TGF-β pathway and repressed genes important for tumorigenesis [30]. The deletion of *FOXN3* gene is recurrent in this study, suggesting that *FOXN3* possibly serves as a novel TSG and contributes to the leukemogenesis. However, subsequent expression study suggested that reduced expression of *FOXN3* was not only presented in AML-M5 but also other subtypes of AML, indicating that the *FOXN3* encodes a myeloid marker rather than a specific leukemogenic protein of AML-M5. The *MLL* gene is reported to be highly associated with AML with complex karyotypes [31]. In this study, it suggests that the amplication of *MLL* frequently co-exists with the deletion of *CDKN2A*. *CDKN2A* encode cyclin-dependent kinase inhibitor, which have emerged as an important effecter in aging as well as a potent tumor suppressor. The main genetic alterations involving *CDKN2A* are deletions by bi- or monoallele or hypermethylation of 5′ GpG island in its promotor [32]–[34]. In AML, hypermethylation of *CDKN2A* has been reported, but deletion of *CDKN2A* is rare [35]–[37]. It is intriguing to reveal the coexistence of *MLL* amplication and *CDKN2A* deletions in AML-M5 and the cooperation of these genes rearrangements in leukemogenesis of AML-M5 needs to be further clarified.

Due to the limitation of case number, no statistical difference was shown between the number of CNAs and clinico-biological parameters in our analysis. More cases are necessary to evaluate the clinical significance of the number of CNAs.

In summary, we genetically defined AML-M5 by karyotype, FISH and array CGH. Array CGH is necessary to complement the traditional cytogenetic analysis in revealing the cryptic genomic alterations in AML-M5. As a subtype of AML, AML-M5 carries both common recurrent CNAs coexisting in AML and novel CNAs in AML-M5. The recurrent CNAs harbor oncogenes or tumor suppressor genes. Further studies are needed to understand the role of these genes in the leukemogenic network of AML.

## Methods

### Patients and clinical data

This research project was approved by both the Ethical Committee of the First Affiliated Hospital of China Medical University and the Institutional Review Board (IRB) at the University of Oklahoma Health Sciences Center (OUHSC) (IRB #13100). Before data collection a written informed consent from was obtained from each participant. Bone marrow samples were collected from 24 adults with AML-M5 (n = 22 at diagnosis, n = 2 at relapse) who referred to the First Affiliated Hospital of China Medical University between May 2009 and December 2010. The patients aged <14 years old were excluded in this study. The diagnosis was made according to the French-American-British (FAB) Cooperative Group criteria [38]. The ratio of males and females is 15∶9 and the median age of patients was 47 years old (range, 15 to 72 years). The average value of blast count in bone marrow was 0.72 (range, 0.40 to 0.98). The clinical information of the patients is summarized in Table S4 in File S1.

### Therapy

Twenty two patients were newly diagnosed and received a single course of daunorubicin plus cytarabine (DA) or idarubicin plus cytarabine (IA) regimen for induction treatment. For the patients who failed to achieve complete remission (CR) or relapsed from CR, salvage therapy was used, including low-dose cytarabine, aclarubicin and granulocyte colony-stimulating factor (G-CSF) (CAG) or fludarabine, cytarabine and G-CSF (FLAG) regimen. Intermediated dose cytarabine (IDAra-C) ± antharcycline, mitoxantrone plus cytarabine (MA) and DA were chosen as postremission therapy for patients achieved CR and it lasted for 6–8 courses. No further therapy was applied to patients remaining in remission after postremission therapy. The details of each regimen were listed in Table S5 in File S1.

### Criteria of response and definition of relapse

For response assessment, bone marrow aspiration was performed between days 21 and 28 after induction therapy. CR was defined as a bone marrow with normal hematopoiesis, blasts in bone marrow less than 5%, granulocyte count ≥1.0×10^9^/L, platelet count ≥100×10^9^/L, no myeloid blasts in the peripheral blood and no extramedullary disease. Patients who did not fulfill the above criteria were defined as unremission. Relapse was defined as the reappearance of more than 5% blasts in bone marrow aspirates or extramedullary leukemia in patients with a previously documented CR.

### Ficoll gradient separation and cryopreservation

Bone marrow mononuclear cells (MNCs) were isolated by Ficoll-paque gradient centrifugation (TAKARA Biotechnology, Dalian, China), aliquoted into fetal calf serum (FCS) with 10% DMSO, and cryopreserved in the liquid phase of a liquid nitrogen tank.

### Preparation of Sample DNA and RNA

Liquid nitrogen-cryopreserved (liquid phase) bone marrow mononuclear cells were washed and recovered by centrifugation. Following that, DNA was extracted with phenol-chloroform, and precipitated with ammonium acetate, ethanol and glycogen. RNA was prepared from approximately 2×10^5^ to 2×10^6^ blasts with Trizol (TAKARA Biotechnology, Dalian, China), and resuspended in 50 μl DEPC-treated water. Complementary DNA was made from ∼20 ng of RNA using the SuperScript III first strand synthesis kit and oligo-dT priming [Promega Corporation, an affiliate of Promega (Beijing) Biotech, China].

### Cytogenetics

Short-term cultures of unstimulated bone marrow samples were prepared and fixed according to our standard laboratory protocols. Karyotype analysis was performed by R banding technique. Chromosome study could not been performed on six bone marrow samples due to the limitation of cell number. The cytogenetic abnormalities were described according to the International System for Human Cytogenetic Nomenclature.

### Array CGH

Array CGH analysis was performed on all DNA samples obtained from 24 patients with AML-M5 following the manufacturer's protocol with minor modifications, (Roche NimbleGen System Inc., Madison, WI). The array consisted of approximately 720,000–mer oligonucleotide probes that spanned both coding and noncoding sequences with average genome coverage of approximately 2 kilobases. A commercially available pooled normal control DNA was used (Promega Corporation, Madison, WI) for reference. The patients and the reference DNA were labeled with either Cyanine 3 (Cy-3) or Cyanine 5 (Cy-5) by random priming (Trilink Biotechnologies, San Diego, CA) and then hybridized to the chip via incubating in the MAUI hybridization system (BioMicro Systems, Salt Lake City, UT). After 40-hours hybridization at 42°C, the slides were washed and scanned using a GenePix 4000B (Molecular Devices, Sunnyvale, CA). NimbleScan version 2.4 and the SignalMap version 1.9 were applied for data analysis (NimbleGen System Inc, Madison, WI). The genomic locations were retrieved from National Center for Biotechnology Information (NCBI) build 36 (hg 18). Frequently affected regions recently detected as copy number polymorphisms were excluded from data analysis according to the Chromosome Number Variation (CNV) database in our lab and genomic variants in human genome (Build 36: Mar. 2006). The genomic data of array CGH are available in NCBI Gene Expression Omnibus (GEO) under accession number GSE53429.

### Fluorescence In Situ Hybridization (FISH)

Confirmatory FISH analysis was performed using different probes. (1) vysis LSI MLL dual color break apart rearrangement probe and vysis LSI CDKN2A (9p21)/CEP9 probe (Abbott Molecular Inc., Des Plaines, IL) on case #18. (2) EVI t(3;3) inv(3) Break, Dual-Color Probe (Kreatech Diagnostics, Amsterdam, TN) on 69 de novo AML patients' bone marrow cells. The FAB subtypes and karyotypes of the patients were showed in Table S2 in File S1. (3) Vysis LSI ETV6(TEL)/RUNX1(AML1) ES Dual Color Translocation Probe (Abbott Molecular Inc., Des Plaines, IL) on the case with ID 37 in Table S2 in File S1.

### Expression of *FOXN3* determined by Quantitative real-time PCR (qRT-PCR)

qRT-PCR was performed to determine the expression of *FOXN3* on bone marrow MNCs of 97 patients with acute leukemia and 16 normal controls who had normal myelograms (Table S3 in File S1). Complementary DNAs were made from ∼20 ng of RNA using the Superscript I II first strand synthesis kit (Invitrogen) and oligo-dT priming. Primers and TaqMan-based probes were purchased from Applied Bio-systems (Applied Biosystems, Beijing, China) and listed in Table S6 in File S1. Duplicate amplification reactions were done with an ABI 7500 Real time PCR System (Applied Biosystems, Foster City, CA, USA) and the data were analyzed by the ABI 7500 system SDS software (1.4 Version).

### Statistical analysis

The Fisher's exact test was employed to assess the association of CNAs numbers per case with clinico-biological parameters. The correlation of the expression levels of *FOXN3* and the subtypes of acute leukemia was analyzed by the student's t-test. All statistical analyses were performed using GraphPad Prism5 and p-value ≤0.05 was considered as statistically significant.

## Supporting Information

File S1 Figure S1, 3q anomaly identified by karyotype and FISH in two of the 69 de novo AMLs. (A)–(F) showed t(3;21) in case with ID 37 and (G)–(I) showed inv(3) in case with ID 65. Arrows indicated the der(3)t(3;21) (A) and der(21)t(3;21) (B) observed by R-banding. FISH with EVI(3q26) probe showed two fusion signals plus an extra red signal (TEL'EVI1) in interphase (C) and translocation of chromosome 3 with a variant breakpoint of *EVI* gene in metaphase (D). Hybridization with TEL/AML1 probe demonstrated an extra red signal in interphase (E) and translocation of chromosome 21 in metaphase (F). (G) Arrow indicated the derivative inv(3) showed by R-banding. FISH with EVI(3q26) probe showed two fusion signals plus an extra red signal (TEL'EVI1) in interphase (H) and inv(3) with a variant breakpoint of *EVI* gene in metaphase (I). Figure S2, The comparisons of *FOXN3* Levels in health control, ALL and AML. The significantly reduced *FOXN3* level was observed between AML and health controls. *: p<0.05. The *FOXN3* level was lower in ALL than health control whereas no significantly difference was observed. Figure S3, The comparisons of *FOXN3* Levels in health control, M5 and non-M5. No significantly difference was observed on the *FOXN3* levels between AML-M5 and non-AML-M5 (p>0.05). Table S1, The results of karyotype and array CGH in 24 AML-M5. Table S2, FAB subtypes, karyotypes and FISH with EVI (3q26) probe analysis on 69 de novo AML patients. Table S3, Clinical information and *FOXN3* expression levels of 97 acute leukemia samples and 16 normal controls. Table S4, The clinical characterizations of 24 cases of AML-M5. Table S5, Drug dose and duration of chemotherapy. Table S6, Primers in qRT-PCR for *FOXN3*.
(7Z) Click here for additional data file.
